# Comparative analysis of the risk of severe bacterial infection and septicemia in adolescents and young adults with treatment-resistant depression and treatment-responsive depression - a nationwide cohort study in Taiwan

**DOI:** 10.1007/s00787-025-02684-y

**Published:** 2025-03-08

**Authors:** Jia-Ru Li, Yu-Chen Kao, Shih-Jen Tsai, Ya-Mei Bai, Tung-Ping Su, Tzeng-Ji Chen, Chih-Sung Liang, Mu-Hong Chen

**Affiliations:** 1 Department of Psychiatry, Far Eastern Memorial Hospital, New Taipei City, Taiwan; 2 Institute of Epidemiology and Preventive Medicine, College of Public Health, National Taiwan University, Taipei, Taiwan; 3 Department of Psychiatry, Tri-Service General Hospital, National Defense Medical Center, Taipei, Taiwan; 4 Department of Psychiatry, Beitou Branch, Tri-Service General Hospital, No. 60, Xinmin Road, Beitou District, Taipei City, 112 Taiwan; 5 Department of Psychiatry, Taipei Veterans General Hospital, Taipei, Taiwan; 6 Department of Psychiatry, College of Medicine, National Yang Ming Chiao Tung University, Taipei, Taiwan; 7 Department of Psychiatry, General Cheng Hsin Hospital, Taipei, Taiwan; 8 Department of Family Medicine, Taipei Veterans General Hospital, Taipei, Taiwan; 9 Institute of Hospital and Health Care Administration, National Yang Ming Chiao Tung University, Taipei, Taiwan; 10 Department of Family Medicine, Hsinchu Branch, Taipei Veterans General Hospital, Hsinchu, Taiwan; 11 Department of Medical Research, Taipei Veterans General Hospital, No. 201, Sec. 2, Shih-Pai Road, Taipei, 112 Taiwan

**Keywords:** Major depressive disorder, Treatment-resistant depression, Treatment-responsive depression, Bacterial infection, Septicemia, Adolescent and young adult

## Abstract

Previous studies have shown an association between depression and increased susceptibility to infection in the general population. However, there has been no prior research specifically examining the relationship between treatment-resistant depression (TRD) and severe bacterial infections (SBI) in adolescents and young adults. This retrospective observational cohort study utilized the Taiwan National Health Insurance Research Database (NHIRD) from 2001 to 2010. It included adolescents (12–19 years of age) and young adults (20–29 years of age) diagnosed with major depressive disorder (MDD), comprising 6958 cases of TRD and 27,832 cases of antidepressant-responsive depression (ARPD). The TRD and ARPD groups were further matched (4:1) by chronological age, age at diagnosis of depression, sex, residence, and family income. The primary outcomes were severe bacterial infections (SBI) and septicemia. Cox regression analysis was conducted to identify the risk of hospitalization due to SBI or septicemia during the follow-up period. Compared with controls, the ARPD group had increased risks of SBI (hazard ratio [HR] with 95% confidence interval [CI]: 3.90, 2.73–5.57) and septicemia (HR, 95% CI: 2.56, 1.34–4.91). Notably, the risks of SBI and septicemia appeared to be further elevated in the TRD group. The TRD group exhibited higher incidences of SBI (HR, 95% CI: 6.99, 4.73–10.34) and septicemia (HR, 95% CI: 2.85, 1.28–6.36) than the control group. Adolescents and young adults with TRD had 6.99-fold and 3.90-fold increased risks of SBI and septicemia compared to individuals without MDD, respectively. Therefore, healthcare providers need to be vigilant when monitoring and implementing preventive measures in this population.

## Introduction

Major depressive disorder (MDD) is a significant mental health concern and a leading cause of disability. It typically begins in adolescence and contributes significantly to the disease burden among young individuals. Studies estimate the prevalence of MDD to be approximately 4–6% among adolescents [1–3]. Moreover, the incidence of depressive symptoms and MDD increases sharply during adolescence [1–3]. The onset of MDD during adolescence is associated with increased illness severity in adulthood, increased psychiatric morbidity, and poor physical health outcomes [4, 5]. Adolescents and young adults with MDD tend to experience significant social and educational difficulties, increasing the risk of long-term psychosocial impairment in adulthood [6–9].

The largest study of adolescents with treatment-resistant depression (TRD), the *Treatment of SSRI-Resistant Depression in Adolescents (TORDIA) Study*, defined TRD in adolescents as those required to have failed treatment with an SSRI and to have been treated for at least 8 weeks [10]. A previous study conducted in Taiwan showed that up to 23% of adolescents with MDD exhibit a poor response to initial antidepressant treatment [11]. Additionally, approximately 2% of these patients had their antidepressant treatment regimens altered two or more times [11]. Resistance to antidepressants in adolescents with MDD is associated not only with psychiatric comorbidities such as anxiety disorders, substance use disorders, and attention deficit hyperactivity disorder, but also with general medical conditions [11, 12]. TRD episodes are associated with significantly increased healthcare resource utilization, including higher rates of all-cause hospitalizations and emergency room visits compared to ARPD MDD episodes [13–15]. They are also nearly three times longer in duration, which substantially contributes to their total costs being 3.5 times higher than those of ARPD MDD episodes in the general population [13]. This increased utilization not only reflects the severity of TRD but also poses a higher risk for physical comorbidities [12, 16]. This prolonged disease burden leads to heightened emotional and financial strain on both patients’ families and caregivers [13, 14].

Given these substantial impacts of TRD, it is crucial to also consider the increased risk of infections that adolescents and young adults face. These individuals experience various transitional stages, during which their health risks and causes of death evolve significantly [17]. According to the *Global Accelerated Action for the Health of Adolescents (AA-HA! )*, infectious diseases are the leading cause of mortality among children aged 10–14 years [17]. While road injuries are the primary cause of death among older adolescents and young adults (15–19 years), infectious diseases remain a significant contributor to mortality within this age group [17]. This increased vulnerability can be attributed to various factors, such as hormonal changes, heightened social interactions, and higher engagement in risk-taking behaviors during this developmental phase [18, 19]. A study revealed a higher incidence of severe bacterial infections (SBI) (Odds Ratio [OR], 95% CI: 1.8, 1.6–2.0) and sepsis (OR, 95% CI: 2.3, 1.1–5.0) among adolescents compared to children, resulting in more frequent hospital admissions [20]. Severe infections can lead to septicemia, characterized by the occurrence of sepsis accompanied by the presence of pathogenic microorganisms within the bloodstream [21]. Septicemia is associated with significant negative consequences such as organ dysfunction, long-term impairment, and high mortality rates [21]. Addressing infections in global public health is thus of significant importance. A Japanese study observed a 25% overall mortality from sepsis in adolescents in intensive care units. Moreover, an alarming estimate of more than 10 million sepsis-related deaths annually highlights the substantial contribution of infections to over 20% of global mortality [22, 23]. Despite the encouraging decrease in the age-standardized incidence of sepsis by 37.0% (95% uncertainty interval [UI] 11.8–54.5) and mortality by 52.8% (47.7–57.5) from 1990 to 2017, addressing the burden of severe bacterial infections (SBI) and septicemia remains a pressing priority [23].

In the current study, using longitudinal data from a large sample population drawn from the Taiwan National Health Insurance Research Database (NHIRD), we investigated the risk of developing SBI and septicemia in adolescents and young adults with MDD, especially TRD. We hypothesized that adolescents and young adults with TRD have a higher risk of SBI and septicemia in later stages of life relative to adolescents and young adults with antidepressant-responsive depression (ARPD), and those without depression.

## Methods

### Data source

The Taiwan NHIRD is audited and released by the National Health Research Institute (NHRI) for scientific and study purposes upon the formal application. In the current study, we linked two datasets of the NHIRD, namely, the specialized dataset of mental disorders and the longitudinal health insurance database, for analysis. The specialized dataset of mental disorders included all medical records of all insured individuals with mental disorders, and thus, was used for identifying participants of the study group in the current study. The longitudinal health insurance database includes all medical records of 3,000,000 insured individuals randomly selected from the entire Taiwanese population (approximately 28,000,000) and was used to identify participants of the control group. The diagnostic codes used were based on the International Classification of Diseases, 9th Revision, Clinical Modification (ICD-9-CM). The NHIRD has been extensively used in many epidemiological studies in Taiwan [24–27]. The data used in this study were extracted from the NHIRD registries from 2000 to 2011 based on the availability of data from this period. This study was approved by the Institutional Review Board of the Taipei Veterans General Hospital. The Taipei Veterans General Hospital Institutional Review Board approved the study protocol and waived the requirement for informed consent because this investigation used de-identified data and no human subject contact was required.

### Study protocol

Adolescents (aged 12–19 years), and young adults (aged 20–29 years), who were diagnosed with MDD (ICD-9-CM codes: 296.2, 296.3) by board-certified psychiatrists and had no prior history of SBI between 2001 and 2010 were included in this study. To specifically identify SBI and improve our diagnostic validity, only the diagnostic codes of bacterial infections in the inpatient dataset were included in the present study. Septicemia was defined using the ICD-9-CM code 038 in the inpatient dataset. In Taiwan, to reduce and control the risk of developing antibiotic-resistant bacteria, hospital infectionists supervise the use of antibiotics in infectious diseases, and cultures of infectious origins are required in clinical practice. These clinical procedures aim to ensure the diagnostic validity of infections and their origins. Patients with MDD were classified into two groups (ARPD and TRD) based on their antidepressant treatment response during 1 year of follow-up following diagnosis [11, 28]. An adequate trial of antidepressant treatment was defined as the use of an antidepressant within its therapeutic dosage range (e.g., fluoxetine ≥ 20 mg/day) for ≥ 60 consecutive days [11, 12]. Patients who remained on a single antidepressant were assigned to the ARPD group, and those who changed the antidepressant treatment regimen two or more times were assigned to the TRD group. The ARPD and TRD groups were further matched (4:1) by chronological age, age at the time of depression diagnosis, sex, residence, and family income. For the control group, participants were also age-, sex-, family income-, and residence-matched (1:4), after eliminating participants that were potential study cases, namely, those who had any diagnostic code for severe mental disorders (ICD-9-CM codes: 295, 296, 300.3, 300.4, and 311) in the database, and those who had been diagnosed with SBI before enrollment. SBI were identified in the inpatient dataset from enrollment until the end of 2011. Additionally, Charlson Comorbidity Index (CCI) and all-cause clinical visits were provided for the study and control cohorts. CCI consisting of 22 physical conditions was also assessed to determine the systemic health conditions of all enrolled individuals [29]. CCI includes myocardial infarction, congestive heart failure, peripheral vascular disease, cerebrovascular disease, dementia, chronic pulmonary disease, rheumatologic disease, peptic ulcer disease, liver disease, diabetes, hemiplegia, paraplegia, renal disease, malignancy, leukemia, lymphoma, and AIDS [29]. In order to avoid the bias from the confounding effect of other infection-related physical comorbidities, we additionally assessed asthma, thyroid disorders, anemia, and congenital anomalies in the present study. Income level (levels 1–3 per month: ≤ 19,000 NTD (New Taiwanese Dollars), 19,001–42,000 NTD, and ≥ 42,001 NTD) and urbanization level of residence (levels 1–5, most to least urbanized) were regarded as the proxies for healthcare availability in Taiwan [30].

### Statistical analysis

For between-group comparisons, the F-test was used for continuous variables and Pearson’s X^2^ test was used for nominal variables, where appropriate. Cox regression models with adjustments for age, sex, level of urbanization, income, and CCI scores were used to investigate the HR with a 95% confidence interval (CI) of SBI and septicemia during the follow-up between groups. Finally, given the hypothesis that depressed patients who responded to antidepressant treatment were less likely to be affected by the inflammatory and immunological dysfunction [31, 32], we investigated the likelihoods of severe bacterial infection and septicemia between patients with TRD and the combined group of patients with ARPD and non-depressed control individuals. A 2-tailed *P*-value of less than 0.05 was considered statistically significant. All data processing and statistical analyses were performed using the Statistical Package for Social Science, version 17 (SPSS; SPSS Inc., Armonk, NY, USA) and Statistical Analysis Software version 9.1 (SAS; SAS Institute, Cary, NC).

### Data availability

The NHIRD was released and audited by the Department of Health and the Bureau of the NHI Program for Scientific Research (https://nhird.nhri.org.tw/). The NHIRD can be obtained through a formal application regulated by the Department of Health and the Bureau of the NHI Program.

## Results

In total, 62,622 adolescents and young adults were included in this study, including 6,958 adolescents and young adults with TRD, 27,832 adolescents and young adults with ARPD, and 27,832 controls (Table 1). The mean age is approximately 22.8 years, with a female predominance of 52.6% of the population (Table 1). Additionally, adolescents and young adults with TRD were more likely to have higher CCI scores and other physical comorbidities than those in the other two groups (*P* < 0.001) (Table 1).

**Table 1:** Demographic characteristics between adolescents and young adults with treatment-resistant and antidepressant-responsive depression

	A. Adolescents and young adults with treatment-resistant depression (*n* = 6958)	B. Adolescents and young adults with antidepressant-responsive depression (*n* = 27,832)	C. Control group (*n* = 27,832)	*p*-value	Post-hoc
Age at depression diagnosis or enrollment (years, SD)	22.84 (4.04)	22.80 (4.01)	22.83 (4.05)	0.646	
Male (n, %)	3301 (47.4)	13,204 (47.4)	13,204 (47.4)	>0.999	
Level of urbanization (n, %)				>0.999	
1 (most urbanized)	1948 (28.0)	7792 (28.0)	7792 (28.0)		
2	2343 (33.7)	9372 (33.7)	9372 (33.7)		
3	908 (13.0)	3632 (13.0)	3632 (13.0)		
4	631 (9.1)	2524 (9.1)	2524 (9.1)		
5 (most rural)	1128 (16.2)	4512 (16.2)	4512 (16.2)		
Income-related insured amount (n, %)				>0.999	
≤ 19,100 NTD/month	1290 (18.5)	5160 (18.5)	5160 (18.5)		
19,001~42,000 NTD/month	2370 (34.1)	9480 (34.1)	9480 (34.1)		
> 42,000 NTD/month	3298 (47.4)	13,192 (47.4)	13,192 (47.4)		
CCI score (SD)	0.97 (1.16)	0.80 (1.03)	0.45 (0.77)	<0.001	A> B> C
Other physical comorbidities (n, %)					
Thyroid disorders	327 (4.7)	953 (3.4)	466 (1.7)	<0.001	A> B> C
Anemia	197 (2.8)	677 (2.4)	398 (1.4)	<0.001	A> B> C
Asthma	435 (6.3)	1389 (5.0)	672 (2.4)	<0.001	A> B> C
Congenital anomalies	90 (1.3)	253 (0.9)	135 (0.5)	<0.001	A> B> C

SD: standard deviation; NTD: New Taiwan dollar; CCI: Charlson Comorbidity Index

During the follow-up duration of 12 years, the incidence of SBI was highest in the TRD group (1.3%), followed by the ARPD group (0.6%) and the control groups (0.1%) (*P* < 0.001) (Table 2). The incidence of septicemia was also highest in the TRD group (0.2%), followed by the ARPD group (0.1%) and the control group (0.0%) (*P* < 0.001) (Table 2). The age at diagnosis of septicemia was significantly different for the three groups (24.00 ± 3.53 in the TRD group, 24.87 ± 3.88 in the ARPD group, and 31.97 ± 4.05 in the control group [*P* < 0.001]) (Table 2).

**Table 2:** Incidence of severe bacterial infection and septicemia between adolescents and young adults with treatment-resistant and antidepressant-responsive depression

	A. Adolescents and young adults with treatment-resistant depression (*n* = 6958)	B. Adolescents and young adults with antidepressant-responsive depression (*n* = 27,832)	C. Control group (*n* = 27,832)	*p*-value	Post-hoc
Incidence of severe bacterial infection (n, %)	122 (1.3)	280 (0.6)	63 (0.1)	<0.001	A> B> C
Incidence of septicemia (n, %)	13 (0.2)	40 (0.1)	12 (0.0)	<0.001	A> B> C
Age at diagnosis (years, SD)	24.00 (3.53)	24.87 (3.88)	31.97 (4.05)	<0.001	A~B< C
Origins of bacteria					
Streptococcus	2 (0.0)	7 (0.0)	5 (0.0)	0.739	
Klebsiella	1 (0.0)	3 (0.0)	5 (0.0)	0.191	
Pseudomonas	1 (0.0)	0 (0.0)	0 (0.0)	0.018	
Hemophilus	1 (0.0)	1 (0.0)	1 (0.0)	0.472	
Staphylococcus	4 (0.1)	14 (0.1)	2 (0.0)	0.008	A~B> C
Mycoplasma	2 (0.0)	4 (0.0)	0 (0.0)	0.050	A> B> C
Chlamydia	0 (0.0)	1 (0.0)	0 (0.0)	0.535	
Meningococcus	0 (0.0)	0 (0.0)	0 (0.0)	n.a.	
Escherichia	8 (0.1)	18 (0.1)	4 (0.0)	0.001	A> B> C
Tuberculosis	5 (0.1)	10 (0.0)	2 (0.0)	0.007	A> B> C
Zoonotic bacterial diseases	0 (0.0)	0 (0.0)	0 (0.0)	n.a.	
Actinomycotic infections	0 (0.0)	0 (0.0)	0 (0.0)	n.a.	
Rickettsioses	0 (0.0)	4 (0.0)	2 (0.0)	0.472	
Syphilis	2 (0.0)	2 (0.0)	0 (0.0)	0.027	A~B> C
Gonococcus	0 (0.0)	0 (0.0)	0 (0.0)	n.a.	
Trichomoniasis	1 (0.0)	4 (0.0)	0 (0.0)	0.135	

SD: standard deviation; n.a.: not available

Adolescents and young adults with TRD had a 6.99 times higher risk (HR, 95% CI: 6.99, 4.73–10.34), and those with ARPD had a 3.90 times higher risk (HR, 95% CI: 3.90, 2.73–5.57) of developing SBI in later life compared to the control group (Table 3). Both the TRD (HR, 95% CI: 2.85, 1.28–6.36) and ARPD groups (HR, 95% CI: 2.56, 1.34–4.91) had an increased risk of septicemia later in life compared with the control group (Table 3).

**Table 3:** Risks of developing severe bacterial infection and septicemia between adolescents and young adults with treatment-resistant and antidepressant-responsive depression*

	HR, 95% CI	
	Severe bacterial infection	Septicemia
Control group	1 (ref)	1 (ref)
Antidepressant-responsive depression group	**3.90 (2.73-5.57)**	**2.56 (1.34-4.91)**
Treatment-resistant depression group	**6.99 (4.73-10.34)**	**2.85 (1.28-6.36)**
Control and antidepressant-responsive depression group	1 (ref)	1 (ref)
Treatment-resistant depression group	**2.61 (2.03-3.37)**	1.48 (0.80-2.75)

HR: hazard ratio; CI: confidence interval; CCI: Charlson Comorbidity Index

*: adjusting for demographic characteristics, CCI, and other physical comorbidities

**Bold type** indicates statistical significance

**Fig. 1:**
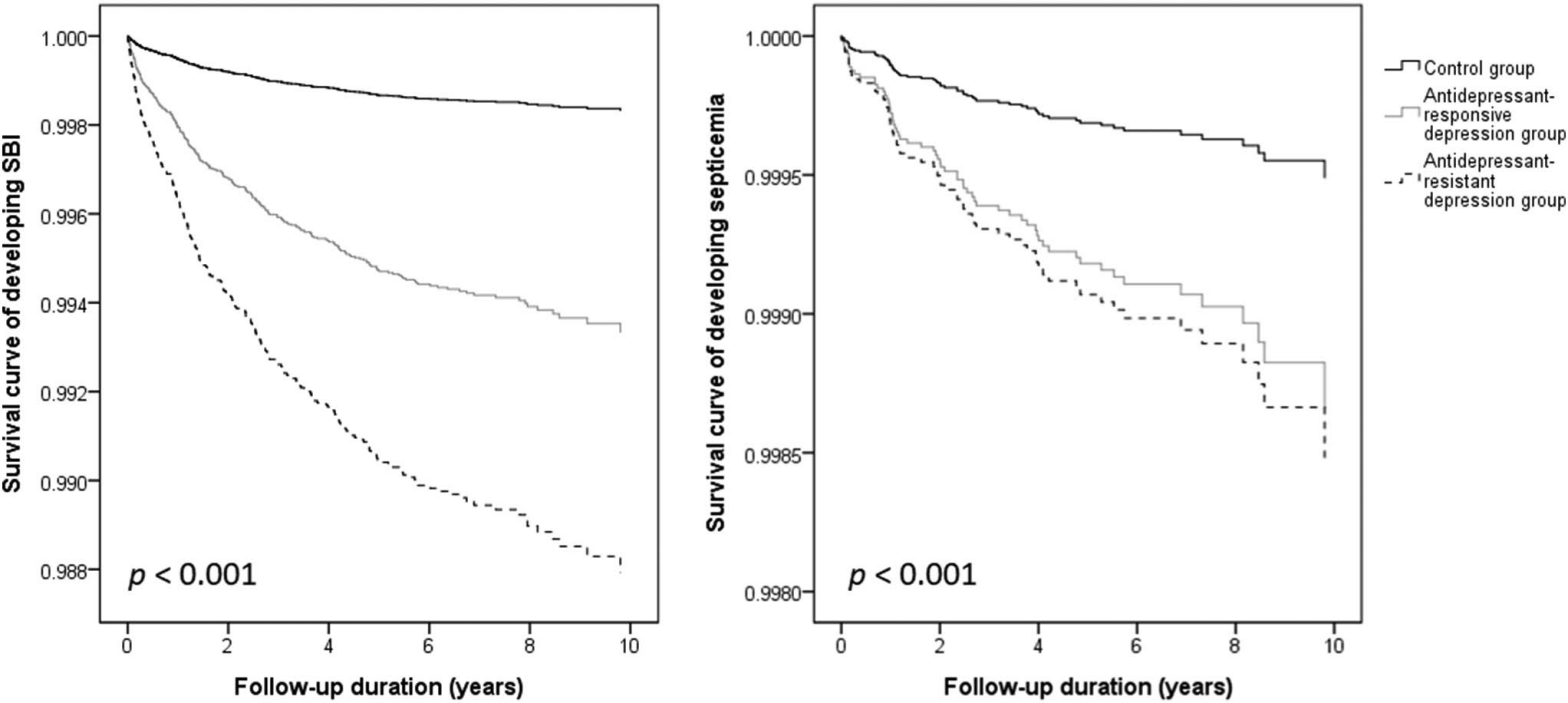
Survival curves of developing severe bacterial infection and septicemia between adolescents and young adults with treatment-resistant and antidepressant-responsive depression. SBI: severe bacterial infection

Fig. 1 shows the Kaplan-Meier curves of SBI and septicemia in the TRD, ARPD, and control groups. The cumulative SBI and septicemia rates during the maximum 12-year follow-up revealed a statistically significant difference among the three groups.

## Discussion

To our knowledge, this study is the first national follow-up study with a large sample size to investigate TRD and SBI among adolescent and young adults. Our research findings suggest that adolescents and young adults with TRD and ARPD have a significantly higher risk of developing SBI and septicemia compared to the control group. Specifically, the risk for SBI was 6.99-fold in those with TRD and 3.90-fold in those with ARPD, while the risk for septicemia was 2.85-fold and 2.56-fold, respectively. The response to antidepressant treatment was identified as an independent factor influencing the development of SBI and septicemia in adolescents and young adults with MDD.

We found that adolescents and young adults with TRD had a higher incidence of SBI and septicemia than those with ARPD and healthy controls. This finding is consistent with those of previous studies. An analysis of a combined dataset consisting of 130,652 participants from the UK Biobank and additional data from Finnish replication cohorts comprising 109,781 participants revealed a link between depression and an increased susceptibility to bacterial infections requiring hospitalization (HR 2.52; 95% CI, 1.99–3.19) [33]. Andersson et al. suggested a correlation between depression and escalated susceptibility to a range of infections, particularly sepsis, which showed the highest risk at approximately 2.39 times higher [34]. Adams et al.. further revealed that college students, aged 18–24 years, who reported not experiencing depression, anxiety, or exhaustion were consistently associated with a lower probability of infectious diseases [35]. Moreover, Hamer et al.. demonstrate a correlation between the levels of depression and anxiety distress symptoms and the risk of mortality due to infectious diseases [36]. Previous research has suggested the possibility of a dose-response relationship between the number of depressive episodes and the occurrence of infections, as well as the severity of symptoms associated with infectious disease mortality [34, 36]. Additionally, even during antidepressant treatment, individuals with depression may be at the highest risk of adverse infection-related outcomes [37]. Consistent with these findings, our study demonstrated that adolescents and young adults with TRD were 6.99 times more likely to develop SBI than controls, with a significantly higher risk compared to the ARPD group, which had a 3.90-fold higher risk than controls.

A previous study suggested that the TRD group may manifest characteristics indicative of more severe depression, as well as more medical comorbidities [38]. The coexistence of TRD and infectious conditions may be influenced by various factors, such as socioeconomic status (SES), shared environmental risk factors, or shared genetic elements [39, 40]. Low SES is independently associated with depression and susceptibility to infections [39, 41, 42]. Our study matched the assessed population based on the level of urbanization of residency, serving as an approximate means to control for potential confounding variables related to SES. Poor personal hygiene can potentially increase vulnerability to infection among adolescents and young adults with TRD. Patients with TRD who neglect self-care could also be at risk of common infections that progress to severe SBI and septicemia. Additionally, heightened instances of risk-taking behaviors, such as injuries, are associated with TRD, which potentially elevate the risk of open wounds and contribute to the development of SBI and septicemia [43, 44]. Moreover, nonadherence to medication may contribute to both TRD and antibiotic resistance, thereby worsening the prognosis and increasing the risk of SBI and septicemia.

Several mechanisms may influence the relationship between TRD and SBI development in adolescents and young adults. First, the association between depression and the risk of infection may be mediated through depression-related immunological changes. Individuals with elevated levels of proinflammatory cytokines may have a greater propensity to exhibit resistance to antidepressant medications, thus, potentially heightening their vulnerability to infections and sepsis [31, 45]. Depression has been linked to the upregulation of inflammatory cytokines and acute-phase reactants, such as interleukin 6, tumor necrosis factor alpha, and C-reactive protein, which are strongly associated with infection [31, 46, 47]. Second, in patients with depression, abnormalities in the hypothalamic-pituitary-adrenal (HPA) axis have been observed. The abnormalities were associated with the development of depression and antidepressant resistance [48–50]. Additionally, dysregulation of the HPA contributes to the development of systemic bacterial infection and sepsis [51, 52]. Third, the gut microbiome plays a key role in the development and progression of various infectious and inflammatory diseases [53]. Growing evidence supports the bidirectional relationship between depression and the gut microbiome. Antidepressant use has been shown to influence the gut microbiome’s composition and function, potentially affecting the brain-gut interaction [54–56]. However, the exact mechanisms underlying the effects of antidepressants on gut microbiome remain unclear. Additionally, a Genome-Wide Association Studies (GWAS) study suggested a link between genetically predicted major depressive disorder and an increased risk of sepsis. This finding supports the hypothesis that these two conditions may share common genetic factors [57].

The results of this study provide further evidence of the role of TRD in infectious diseases among adolescents and young adults. The strengths of our study include a large sample size and, a database-based longitudinal follow-up. This study also has some limitations. First, the data used in this study were extracted from the NHIRD registries from 2000 to 2011, which could be seen as a limitation given the recent developments in treatments for TRD. However, by focusing on earlier data, our study provides a more consistent view of the relationship between TRD and SBI, as it reduces the potential influence of newer interventions. Second, the TRD data captured in the NHIRD registries are based on diagnoses made in psychiatric treatment settings. Although unwilling to seek medical care or treatment can make it more difficult to observe conditions and may result in the underreporting of depression, this study includes a large sample size that covers almost all adolescents and young adults in Taiwan. Third, the incidence of septicemia in our sample was low. Consequently, despite significant HR, the actual risks remained remarkably low in each of the three groups. Furthermore, the strata for certain bacteria were not fully represented due to limited sample sizes. Fourth, SBI do not commonly occur in adolescents and young adults, except in those with severe physical diseases, such as severe autoimmune diseases. Therefore, we adjusted for the CCI, which includes 22 severe physical conditions, and additionally accounted for some possible physical comorbidities.

## Conclusions

This study found that individuals with TRD have a higher incidence of SBI and septicemia than those with ARPD and controls. Our findings highlight the need for clinicians to remain vigilant regarding adolescents and young adults with TRD, as they are at an increased risk of developing SBI and septicemia later in life. These results underscore the need for appropriate monitoring and prevention of severe infections in this population. Further research is needed to examine the link between TRD, SBI, and septicemia.

## Data Availability

The NHIRD was released and audited by the Department of Health and the Bureau of the NHI Program for Scientific Research (https://nhird.nhri.org.tw/). The NHIRD can be obtained through a formal application regulated by the Department of Health and the Bureau of the NHI Program.
